# Horizontal gene cluster transfer increased hallucinogenic mushroom diversity

**DOI:** 10.1002/evl3.42

**Published:** 2018-02-27

**Authors:** Hannah T. Reynolds, Vinod Vijayakumar, Emile Gluck‐Thaler, Hailee Brynn Korotkin, Patrick Brandon Matheny, Jason C. Slot

**Affiliations:** ^1^ Department of Plant Pathology The Ohio State University 2021 Coffey Road Columbus Ohio 43210; ^2^ Department of Biological & Environmental Sciences Western Connecticut State University 181 White St. Danbury Connecticut 06810‐6826; ^3^ Ecology & Evolutionary Biology University of Tennessee 334 Hesler Biology Building Knoxville Tennessee 37996‐1610

**Keywords:** Agaricales, evolutionary genomics, fungi, horizontal gene transfer, molecular evolution, phylogenomics, psychedelic, secondary metabolite

## Abstract

Secondary metabolites are a heterogeneous class of chemicals that often mediate interactions between species. The tryptophan‐derived secondary metabolite, psilocin, is a serotonin receptor agonist that induces altered states of consciousness. A phylogenetically disjunct group of mushroom‐forming fungi in the Agaricales produce the psilocin prodrug, psilocybin. Spotty phylogenetic distributions of fungal compounds are sometimes explained by horizontal transfer of metabolic gene clusters among unrelated fungi with overlapping niches. We report the discovery of a psilocybin gene cluster in three hallucinogenic mushroom genomes, and evidence for its horizontal transfer between fungal lineages. Patterns of gene distribution and transmission suggest that synthesis of psilocybin may have provided a fitness advantage in the dung and late wood‐decay fungal niches, which may serve as reservoirs of fungal indole‐based metabolites that alter behavior of mycophagous and wood‐eating invertebrates. These hallucinogenic mushroom genomes will serve as models in neurochemical ecology, advancing the (bio)prospecting and synthetic biology of novel neuropharmaceuticals.

**Impact Statement**

The rate of horizontal gene transfer (HGT) between species of microorganisms is thought to be higher for genes located in gene clusters, which often encode all of the enzymatic, regulatory, and transport‐related steps required for a metabolic pathway to function in a single genomic locus. Such clusters may enhance the evolvability of fungi by facilitating the rapid loss or gain of multigene traits such as the production of bioactive molecules. Although developmentally complex mushroom‐forming fungi are thought to experience little HGT compared with morphologically simpler fungi, a scattered distribution of the hallucinogenic molecule psilocybin among diverse “magic” mushrooms led us to hypothesize that its biosynthetic pathway has been dispersed by HGT of a gene cluster. To test our hypothesis, we sequenced the genomes of three distantly related hallucinogenic mushroom species for comparison with closely related, nonhallucinogenic species. We identified a homologous multigene cluster in each hallucinogenic species by searching for clustering among all genes with a psilocybin‐like distribution among mushroom species. The enzymatic functions of genes within this cluster were confirmed here and in another concurrent study, and phylogenetic analyses support HGT of the cluster between divergent dung decomposers in the genera *Psilocybe* and *Panaeolus*, a first for mushroom‐forming fungi. Bioactive molecules like psilocybin are often presumed to have niche‐specific roles, but the ecological contexts in which they evolved are rarely known. We found that distantly related dung‐ and wood‐decay fungi have less variation in their genome content compared to close relatives in alternative niches, suggesting that this content is shaped in part by shared ecological pressures. Coupled with the inheritance patterns of the psilocybin cluster, these data support the hypothesis that psilocybin production is part of a larger adaptive strategy to dung and late wood‐decay niches, which harbor abundant invertebrates that eat or compete with fungi. We speculate that neuroactive compounds like psilocybin that target broadly conserved neurotransmitter receptors may have evolved as a strategy to influence arthropod activity in these niches, and that fungi within these niches could be further sources of neuroactive molecules.

Secondary metabolites are small molecules that are widely employed in defense, competition, and signaling among organisms (Raguso et al. 2015). Due to their physiological activities, secondary metabolites have been adopted by both ancient and modern human societies as medical, spiritual, or recreational drugs. Psilocin is a psychoactive agonist of the serotonin (5‐hydroxytryptamine, 5‐HT) ‐2A receptor (Halberstadt and Geyer 2011) and is produced as the phosphorylated prodrug psilocybin by a restricted number of phylogenetically disjunct mushroom forming families of the Agaricales (Bolbitiaceae, Inocybaceae, Hymenogastraceae, Pluteaceae, Fig. 1A) (Allen 2010; Dinis‐Oliveira 2017). Hallucinogenic mushrooms have a long history of religious use, particularly in Mesoamerica, and were a catalyst of cultural revolution in the West in the mid‐20th century (Nyberg 1992; Letcher 2006). Psilocybin was structurally described and synthesized in 1958 by Albert Hoffman (Hofmann et al. 1958), and a biosynthetic pathway was later proposed based on the transformation of labeled precursor molecules by *Psilocybe cubensis* (Agurell and Nilsson 1968). However, prohibition since the 1970s (21 U.S. Code § 812—schedules of controlled substances) has limited advances in psilocybin genetics, ecology, and evolution. There has been a recent resurgence of research on hallucinogens in the clinical setting; brain state imaging studies of psilocin exposure have identified changes in neural activity and interconnectivity that underlie subjective experiences, and therapeutic trials have investigated psilocybin's potential for treating major depression and addictive disorders (Griffiths et al. 2011; Carhart‐Harris et al. 2012; Petri et al. 2014; Carhart‐Harris et al. 2016; Johnson et al. 2017). Although the ecological roles of psilocybin, like most secondary metabolites, remain unknown, psilocin's mechanism of action suggests metazoans may be its principal targets.

**Figure 1:**
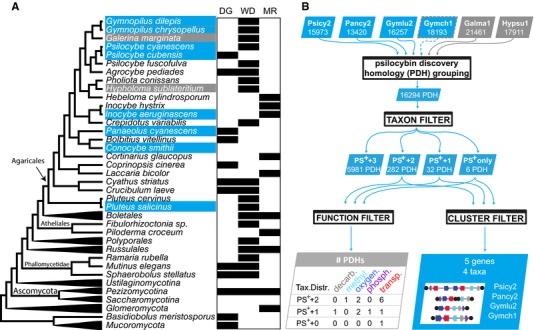
Rationale and approach to search for psilocybin gene clusters. (A) *The patchy distribution of psilocybin in Agaricales from different fungal lifestyles*. Psilocybin (PS) production has a limited phylogenetic distribution among Agaricales fungi, and is associated with ecological lifestyles (dung decay [DG], wood decay [WD], and mycorrhizal [MR]) with similarly spotty distributions. (B) *Pipeline for psilocybin gene cluster discovery*. Protein models from PS^+^ (blue shading) and matched PS^−^ (gray shading) genomes (Psicy2 = *Psilocybe cyanescens*; Pancy2 = *Panaeolus cyanescens;* Gymlu2 = *Gymnopilus dilepis*; Gymch1 = *Gymnopilus chrysopellus*; Galma1 = *Galerina marginata*; Hypsu1 = *Hypholoma sublateritium*) were sorted into psilocybin discovery homolog groups (PDHs), which were filtered by taxon representation. Gymch1 was retroactively inferred PS^+^ (dotted gray outline). Four taxon filters allow for 0, 1, 2, or 3 PS^−^ genomes per PDH. Taxonomically filtered PDHs contained few candidates for decarboxylation, methylation, oxygenation, phosphorylation, and transport‐related functions. A single cluster of taxonomically filtered PDHs was identified in each of four genomes (two partial clusters in *Pa. cyanescens*), and corresponded to expected functions identified in the PS^+^ +1 PDH set.

A common feature of fungal secondary metabolite biosynthesis is the organization of most or all of the required anabolic, transport, and regulatory genes in gene clusters. Gene clusters are often discontinuously distributed among fungal taxa, partly due to horizontal gene transfer (HGT) among species with overlapping ecological niches (Gluck‐Thaler and Slot 2015). The limited phylogenetic distribution of psilocybin (Fig. 1A), coupled with the requirement for multiple enzymatic steps for its biosynthesis (in order: tryptophan‐decarboxylation, tryptamine‐4‐hydroxylation, 4‐hydroxytryptamine O‐phosphorylation, and *N*‐methylation; Fricke et al. 2017) suggested that the psilocybin pathway might have dispersed via HGT of a gene cluster. We therefore predicted that the genetic mechanism for psilocybin biosynthesis would be identified in searches for gene clusters with a common phylogenetic history and distribution restricted to psilocybin producing (PS^+^) mushrooms.

Here, we present the findings of a phylogenomic investigation of hallucinogenic mushroom genomes. We sequenced three known PS^+^ mushrooms (*Psilocybe cyanescens*, *Gymnopilus dilepis*, and *Panaeolus cyanescens*) representing diverse lineages, searched for clustered genes that were simultaneously associated with functions and phylogenetics of PS production, and converged on a single gene cluster that shows signatures of HGT among the sequenced fungi (Fig. 1B and C). We then confirmed the chemical function and specificity of the presumed first step in the PS pathway (conversion of tryptophan to tryptamine). We assessed further evidence of HGT within a dung environment, and investigated the ecological trends in Agaricales genome content, to elucidate the ecological context in which the PS cluster transfer may have taken place. Together, our work suggests that shared environmental selection pressures may have favored the transfer of the PS gene cluster among hallucinogenic fungi, and provides a methodological roadmap for the future discovery of novel fungal pharmaceuticals.

## Results and Discussion

### IDENTIFICATION OF PSILOCYBIN GENE CLUSTERS IN THREE GENERA BY WHOLE GENOME SEQUENCING

We identified candidate psilocybin genes by sequencing three diverse PS^+^ mushroom homokaryon genomes—*Ps. cyanescens*, *Pa*. ( = *Copelandia*) *cyanescens*, and *Gy*. *dilepis* (Table 1, GenBank MG548652‐MG548659), then comparing them to three related mushrooms not known to produce psilocybin (PS^−^): *Galerina marginata*, *Gymnopilus chrysopellus*, and *Hypholoma sublateritium*. Of the 37 PDHs that we identified to be consistent with a PS^+^ distribution among these taxa, only five genes were clustered, all in PS^+^ genomes. We retroactively designated *Gy. chrysopellus*, potentially PS^+^ because it possesses a cluster identical to *Gy. dilepis*, which is not surprising given inconsistent identifications, and geographical variation among *Gymnopilus* spp. phenotypes. Predicted functions of the five clustered genes were also consistent with psilocybin biosynthesis and metabolite transport, and were putatively designated tryptophan decarboxylase (PsiD), psilocybin‐related *N*‐methyltransferase (PsiM), psilocybin‐related hydroxylase (PsiH), psilocybin‐related phosphotransferase (PsiK), and psilocybin‐related transporter (PsiT). The orthologs of these enzymes shared 75–95% sequence similarity with those from a concurrently discovered psilocybin gene cluster in *Ps. cubensis* (Fricke et al. 2017), so we have adopted the same naming conventions here.

**Table 1:** Genome assembly and annotation of psilocybin‐producing mushrooms

	Accession	Length (nt)	Scaffolds	Contigs	Average depth of coverage (*x*)	N50 (nt)	Complete BUSCOs (%)	Total proteins	Decarboxylases/PSD^1^	P450's^1^	Methyltransferases/DUF890 domain—proteins^1^	Kinases/phosphotransferases/PDH term 0PNAW^1^	MFS^1^
*Gymnopilus dilepis* 2	SAMN07169108	47,177,497	8,423	10,681	16.5	33,540	73.43	16,257	28/9	151	89/4	275/29/1	37
*Panaeolus cyanescens*	SAMN07166494	44,965,162	9,521	11,850	25.7	32,751	75.66	13,420	28/8	148	91/2	267/16/2	34
*Psilocybe cyanescens*	SAMN07169033	53,483,841	18,721	38,006	44.7	46,250	72.18	15,973	38/17	178	102/2	298/23/1	44

^1^Functional category of PS genes as annotated in Eggnog.

^2^ *Gy. dilepis* TENN071165 ( = *Gy. aeruginosus* sensu L. R. Hesler) was isolated from oak sawdust in Knoxville, Tennessee October 4, 2013. *Pa. cyanescens* and *Ps. cyanescens* basidiospores were supplied by The Spore Works, Knoxville.

### CONFIRMATION OF PSILOCYBIN GENE CLUSTER ENZYME ACTIVITY

**Figure 2:**
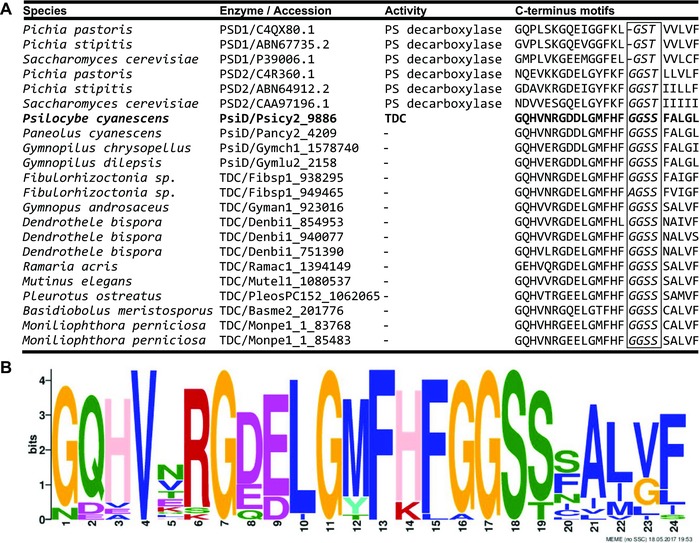
Substrate specificity and C‐terminal sequence signatures of tryptophan decarboxylases (TDCs). (A) Table and sequence alignment of PsiD and other TDC homologs, previously characterized phosphatidylserine decarboxylase (PSD) type I and II enzymes, and closely related fungal TDC‐like proteins indicating conserved C‐terminal residues (386–409). The unique GGSS sequence in the conserved C‐terminal motif is outlined. (B) MEME‐derived sequence logo of conserved C‐terminal residues in fungal homologs of PsiD.

To confirm gene cluster function, we profiled the enzymology of heterologously expressed full‐length cDNAs of *Ps. cyanescens* PsiD and PsiK in bacterial expression systems, and assayed their activities by LC‐MS/MS analyses. We determined that PsiD, the first committed step in the reaction and the only one not producing a drug‐scheduled compound, has specific decarboxylase activity on tryptophan. PsiD reactions produced tryptamine, identified at the characteristic *m*/*z* 144.1 [M + H]^+^, (Fig. S1; Supporting Information Data 1). PsiD did not decarboxylate phenylalanine, tyrosine, or 5‐hydroxy‐l‐tryptophan (5‐HTP) under the same conditions. We note that PsiD is similar to type II phosphatidylserine decarboxylases (PSDs), but has no significant sequence similarity with a pyridoxal‐5’‐phosphate‐dependent decarboxylase recently characterized in *Ceriporiopsis subvermispora* as specific for l‐tryptophan and 5‐HTP (Kalb et al. 2016). A unique GGSS sequence in a conserved C‐terminal motif (Fig. 2), suggests tryptophan decarboxylation is a previously unknown derived function among PSDs (Wriessnegger et al. 2009; Choi et al. 2015). We detected no activity of PsiK on 5‐HT or 4‐hydroxyindole (4‐HI) as alternatives to the psilocin substrate, possibly due to requirements for the 4‐hydroxyl and the methylated amine groups of psilocin. Our further characterization of PsiK and other enzymes was prevented by regulatory restrictions on the possession of substrates and products. However, a recent study has characterized complete biosynthesis of psilocybin by the homologous *Ps. cubensis* cluster (Fricke et al. 2017), serendipitously confirming the function of the gene clusters presented here. The pathway inferred by Fricke et al. (2017) proceeds as follows: tryptophan decarboxylation, tryptamine 4‐hydroxylation, 4‐hydroxytryptamine O‐phosphorylation, and sequential *N*‐methylations to produce psilocybin without a psilocin intermediate. This pathway was unexpected, because previous isotopic studies suggested tryptamine *N*‐methylation precedes hydroxylation of dimethyltryptamine and O‐phosphorylation of psilocin (Agurell and Nilsson 1968). Gene duplications among the clusters we have identified could suggest alternate or reticulated pathways also exist.

### HORIZONTAL TRANSFER OF A PSILOCYBIN GENE CLUSTER

**Figure 3:**
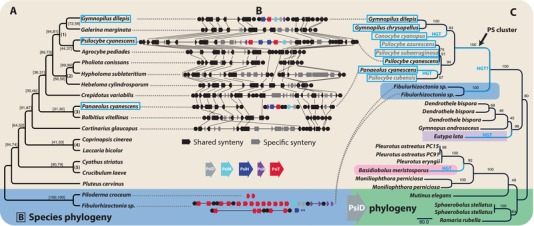
The evolution of psilocybin. (A) Phylogenomic tree of Agaricales (tan shading) with Atheliales (blue shading) outgroup. Support values = (internode certainty, tree certainty). Psilocybin‐producing taxa are indicated by a blue outline box. (B) PS locus synteny relative to *Psilocybe cyanescens* version 2 scaffold 5617 and *Galerina marginata* version 1 scaffold 9. PS clusters consist of a tryptophan decarboxylase (PsiD), one to two P450 monooxygenases (PsiH), a methyltransferase (PsiM), a phosphotransferase (PsiK), and one to two MFS transporters (PSiT). (C) RAxML phylogeny of TDC indicating putative HGT branches; *Eutypa lata* is in Xylariales (Ascomycota, violet shading), an order correlated with absence of termites in coarse woody debris (Kirker et al. 2012), with members that produce a white rot of wood; *Basidiobolus meristosporus* (Zoopagomycota, pink shading) is commonly associated with amphibian dung and arthropods; members of the Phallomycetidae (green shading) commonly associated with dung, decayed wood, and/or insect spore dispersal. Gray taxon names = PCR sequences, black = whole genome. Support is percent of 100 ML bootstraps. **PsiH exists in high copy number in *Fibulorhizoctonia* sp. for which 54 similar PsiH gene homologs are not shown here. Phylogenies of all psilocybin genes are in Figure S2A–E.

Phylogenetic analyses of PS homologs from a local database of 618 fungal proteomes yielded congruent gene tree topologies with respect to PS^+^ taxa, and clades of clustered PS genes from all gene trees excluded the PS^−^ taxa in the database, suggesting the clustered genes are coordinately inherited (Figs. 3 and S2A–E). The gene trees also suggest HGT of the cluster from *Psilocybe* to *Panaeolus* and HGT of most PS genes between Atheliaceae and Agaricaceae when compared to a phylogenomic tree of related Agaricales (Fig. 3). The direction of the latter HGT is ambiguous, and not strongly supported by all five genes. Analyses with additional PsiD and PsiK amplicon sequences retrieved by degenerate PCR of unsequenced *Psilocybe* and *Conocybe* genomes (Supporting Information Data 1, GenBank Accessions MG548652‐MG548659) suggest the dung fungus *Ps. cubensis* vertically inherited the cluster, and *Pa. cyanescens* acquired the cluster from *Psilocybe* sp., possibly from a dung‐associated lineage. Alternative hypotheses of vertical inheritance in these lineages were rejected; constrained topologies that exclude *Pa. cyanescens* and *Conocybe smithii* (AU test, *P* = 0.004) or *Pa. cyanescens* alone (*P* = 0.036) from putative donor clades were rejected (Supporting Information Data 1). Furthermore, a PsiD gene tree‐species tree reconciliation model allowing duplication, HGT, and loss (six events: *D* = 1, HT = 3, *L* = 2) is more parsimonious than a model that only allows duplication and loss (28 events: *D* = 3, *L* = 25) (Fig. S3). PS gene orthologs were not detected in *Ps. fuscofulva*; *Ps. fuscofulva* is sister to the rest of the genus, a pattern consistent with the ancestor of *Psilocybe* being PS^−^ (Borovička et al. 2015). Conservation of synteny flanking the *Ps. cyanescens* PS cluster (Fig. 3B) suggests it may have been recently acquired in *Psilocybe* as well, or alternatively lost as a unit in close relatives. A genome wide scan did not identify any additional HGTs of genes or clusters between *Psilocybe* and *Panaeolus* (Fig. S4); however, additional supported HGTs from Agaricales to distant fungal lineages (Fig. 3C) suggest the constituent gene families may have experienced similar ecological distribution prior to the origin of the psilocybin gene cluster.

Recent studies have suggested HGT is pervasive in the fungi, especially among lifestyle‐associated genes (Wisecaver et al. 2014; Gluck‐Thaler and Slot 2015), and may occur along “highways” (frequent partners in gene exchange) that could correspond to shared environments (Szöllősi et al. 2015; Qiu et al. 2016). However, HGT has been found to be rare in Basidiomycota compared to Ascomycota (Wisecaver et al. 2014), suggesting the transfer of the PS cluster may have provided a significant fitness benefit to the recipient, and is to our knowledge, the first report of HGT of a secondary metabolite gene cluster between lineages of mushroom‐forming fungi (Agaricomycetes). A number of secondary metabolism gene cluster HGT events have been previously reported in Ascomycota, where gene clusters have been much more frequently identified than in Basidiomycota; however, any causal associations among rates of gene clustering, rates of HGT, and the strength of selection among fungal lineages remain largely uninvestigated (Slot 2017).

### ECOLOGICAL DRIVERS OF PSILOCYBIN GENE CLUSTER EVOLUTION

**Figure 4:**
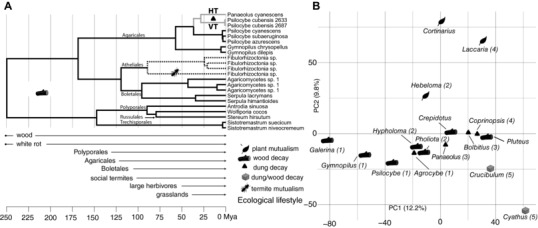
Patterns of ecological diversification of PS genes and Agaricales genomes. (A) Ultrametric representation of the PsiK phylogeny, with root age hypothetically fixed to align ecological transitions with Earth history events. PsiK ecological states are indicated by branch shading (black = wood decay, dashed = termite mutualism, gray = dung decay). HGT and VGT indicate horizontal transfer or vertical transmission of PsiK in the dung environment. (B) Ordination of Agaricales genome content, with species ecology indicated. Numerals correspond to clades in Figure 3A.

Recent studies suggest that ecology can select for both genome content (Ma et al. 2010; de Jonge et al. 2013) and organization in eukaryotes through both vertical and horizontal patterns of inheritance (Holliday et al. 2015; Kakioka et al. 2015). The phylogenies of PS genes suggest they originally served roles in the wood‐decay niche among fungi, and more recently emerged through both vertical and horizontal transfer in dung‐decay fungi (Figs. 3C and 4A). Horizontal transfer and retention of PS clusters are evidence of selection on the PS pathway in the recipient lineage, as secondary metabolite clusters are generally unstable in fungal genomes (Reynolds et al. 2017). In addition to similar ecological pressures, similar genome content among wood and dung‐decaying fungi may also reflect the ecological diversification of Agaricomycetes that accompanied major geological transformations (Fig. 4A). For example, the emergence of true wood opened a massive saprotrophy niche space in the upper Devonian (380 Mya), in which the Agaricomycetes diversified with the aid of key enzymatic innovations (Floudas et al. 2012). The subsequent radiation of herbivorous megafauna during the Eocene approximately 50 MYA (MacFadden 2000) and the spread of grasslands 40 MYA (Retallack 2001) expanded the mammalian dung niche space in which invertebrates and fungi competed. These changes parallel the repeated emergence of dung‐specialization from plant‐decay ancestors in the radiation of *Psilocybe* and other Agaricales lineages (Ramirez‐Cruz et al. 2013; Tóth et al. 2013). Late stage wood‐decay fungi like *Psilocybe* spp. likely harbor genetic exaptations for lignin tolerance/degradation, and competition with invertebrates and prokaryotes; thus acquisition of particularly adaptive functions by other fungi (e.g., *Panaeolus*) through HGT may have further facilitated additional transitions to dung saprotrophy.

Furthermore, ordination of 10,998 Agaricales gene homology groups (AGHs) identified two principal components (PCs) that describe 22% of the variation in gene content among 16 Agaricales genomes (Fig. 4B). Discrimination of genome composition along PC1 appears to reflect phylogenetic differences, whereas discrimination along PC2 parallels ecological differences between plant mutualists and other fungi. However, PC2 does not discriminate between dung and wood‐decay fungi. The functions of AGHs most associated with each PC are consistent with this interpretation. All eight metabolism‐related processes in the COG classification system are overrepresented in PC2 AGHs, but only one in PC1 AGHs (Supporting Information Data 1). The grouping of several divergent lineages of wood‐ and dung‐decay fungi to the exclusion of close ectomycorrhizal relatives along PC2 may reflect similar selective pressures on genome composition in the decayed wood and dung environments, from recalcitrant plant polymers like lignin and invertebrate predation (Rouland‐Lefèvre 2000). However, a small number of AGHs exclusive to either wood‐ or dung‐associated fungi (Supporting Information Data) are consistent with ecological specialization within each guild. Wood‐specific genes include functions in lignin degradation (e.g., peroxidase, isoamyl alcohol oxidase) and carbohydrate transport, whereas dung‐specific genes have functions in bacterial cell wall degradation (e.g., lysozyme), hemicellulose degradation (e.g., endo‐1,4‐beta‐xylanase, alpha‐l‐arabinofuranosidase), and inorganic phosphate transport. Niche‐specific genes are largely consistent with vertical inheritance; however, analyses support HGT of a single ferric‐reductase‐like gene (pfam01794, pfam00175) likely involved in iron uptake, to *Coprinopsis* and *Panaeolus* from dung‐associated Ascomycota (Fig. S2F, Supporting Information Data).

Psilocybin neurological activity, coupled with HGT and retention in lineages that colonize dung and/or decayed wood, which are rich in both mycophagous and competitor invertebrates (Rouland‐Lefevre 2000), suggest that psilocybin may be a modulator of insect behavior. Psilocybin and/or the related aeruginascin have also been identified in the lichenized agaric, *Dictyonema huaorani* (unconfirmed), and in the ectomycorrhizal genus *Inocybe* (Kosentka et al. 2013; Schmull et al. 2014). PS distribution in *Inocybe* is complementary to that of the acetylcholine mimic, muscarine, which could suggest alternative strategies and pressures to manipulate animal behavior beyond the dung‐ and wood‐decay niches. Neurotransmitter mimics may provide advantages to fungi by interfering with the behavior of invertebrate competitors for woody resources (Hunt et al. 2007), especially social insects, like termites, which emerged ∼137 Mya, because they rely on the coordinated activities of multiple castes (Genise 2017). Alternative serotonin receptor 5HT‐2A antagonists have been shown to inhibit feeding in *Drosophila* (Gasque et al. 2013). It is thus intriguing that PsiH and PST have experienced massive gene family expansion by gene duplication in *Fibulorhizoctonia* sp., which produce termite egg‐mimicking sclerotia in an ancient mutualistic relationship with *Reticulitermes* termites (Matsuura 2005). Although neurotransmitter agonists are not known to mediate this symbiosis, insect predatory fungi (i.e., *Cordyceps* spp.) use neurotransmitter analogs to influence the behavior of infected insects (de Bekker et al. 2014), and a number of repellents and toxins in wood‐decay fungi inhibit xylophagy and mycophagy by termites (Rouland‐Lefèvre 2000).

The identification of genes underlying PS biosynthesis is an important advance in the field of neurochemical ecology, with both social and medical applications. The sequences of the first *Psilocybe* and *Panaeolus* genomes presented here and by Fricke et al. (2017) will be important resources for the prospecting of novel neurotropic natural products (Rutledge and Challis 2015). The discovery that a psilocybin gene cluster has been horizontally transferred and subsequently maintained among the invertebrate‐challenged environments of dung and late wood‐decay suggests these niches may be reservoirs not only of new antibiotics (Bills et al. 2013), but also novel neuroactive prodrugs or pharmaceuticals.

## Materials and Methods

### ISOLATION OF MONOSPOROUS ISOLATES

Spores from selected specimens were collected from spore prints, diluted in water and cultured on malt extract agar (MEA) to obtain homokaryotic strains. Each plate was observed for several days (germination time varies depending on species, but generally germination was seen within the first week) under a dissecting scope. Upon germination, the germinating spore was isolated onto a new MEA dish via fine‐tipped forceps. After sufficient mycelial growth, a small, outer‐portion of mycelium was removed and stained with Phloxine B and 5% potassium hydroxide (KOH) and viewed under a compound scope in search of hyphal clamp connections, which would indicate a dikaryotic condition. Because clamps were not found, strains were assumed to be homokaryotic; however, some species do not produce clamps, therefore homokaryotism could not be completely verified until sequencing. A section of the presumed homokaryotic mycelium was placed into a liquid media of potato dextrose broth (PDB) to produce sufficient tissue for genome sequencing.

### GENOME SEQUENCING, ASSEMBLY, AND ANNOTATION

DNA was extracted using the protocol from Hughes et al. (2013). The ribosomal rRNA region ITS was amplified and sequenced using protocols from Birkebak et al. (2013) and ITS sequences were compared to GenBank sequences by BLASTN to predetermine the species and ensure that a contaminant was not extracted before proceeding to entire genome sequencing. Genomic DNA was sequenced using the TruSeq DNA Library Prep kit (Illumina, San Diego, CA) for sequencing with Illumina MiSeq as 2 × 300 bp paired‐end reads to between ∼16× and 44× coverage. The resulting reads were trimmed and error‐corrected using Trimmomatic version 0.32 (Bolger et al. 2014) and SOAPdenovo2 EC version 2.01, then assembled in SOAPdenovo version 4.21 (Luo et al. 2012). Gaps were closed with GapCloser version 1.12 from the SOAPdenovo2 package. Genome assembly completeness was assessed with the fungal dataset in BUSCO version 1.22 (Simão et al. 2015), and coverage depth was calculated in samtools version 0.1.19 (Li et al. 2009) as the average depth of the error‐corrected reads aligned with BWA‐MEM version 0.79a (Li and Durban 2009) to the resulting assemblies.

The genomes were then annotated using a Maker2 version 2.31.8 (Holt and Yandell 2011) pipeline incorporating gene predictions from SNAP (released November 29, 2013) (Korf 2004), Augustus version 2.5.5 (Stanke et al. 2003–2008), and GeneMark‐ES Suite version 4.01 for fungi (Ter‐Hovhannisyan 2008; Borodovsky and Lomsadze 2011). Each psilocybin‐producing mushroom was paired with a closely related mushroom genome for protein homology evidence as follows: *Ga. marginata* proteome for *Ps. cyanescens; Gy. chrysopellus* proteome for *Gy. dilepis; H*. *sublateritium* proteome for *Pa. cyanescens*. Augustus was trained using BUSCO gene predictions for the first Maker2 iteration (settings: protein2genome = 1; max_dna_len = 100000; min_contig = 1000; pred_flank = 150; AED_threshold = 0.7; always_complete = 0; split_hit = 4000; clean_up = 1). The first Maker2 iteration provided the hmm training set for Augustus and SNAP for the second, more stringent Maker2 iteration (altered settings: protein2genome = 0; pred_stats = 1; min_protein = 30; alt_splice = 1; always_complete = 1). In both iterations, repeat regions were masked prior to annotation based on the RepBase 20.01 fungirep database in RepeatMasker version 4.0.1 (Smit et al. 2013–2015). Functional annotations of all predicted proteins were performed using eggNOG‐mapper (version 0.99.1) with fungal‐specific orthology data (Huerta‐Cepas et al. 2015; downloaded April 20, 2017), with options “use all orthologs” and “use non‐electronic terms,” and with sequence searches carried out by HMMER version 3.1b2 (Eddy 2011, http://www.hmmer.org).

### COMPARATIVE GENOMICS ANALYSES

#### Targeted gene cluster discovery

We identified 16,294 orthologous groups of proteins in a six‐proteome dataset using OrthoMCL version 1.4 (Li et al. 2003) using e‐value cutoff 1 × 10^−4^, and then sorted the 6300 found in all three PS^+^ genomes according to the number of taxa containing them (Fig. 1). After removing the 5981 orthologous proteins found in all six proteomes, HMMER version 3.1b2 was used with a 1 × 10^−4^ cutoff to search a fungal‐specific ortholog database (Huerta‐Cepas et al. 2015; downloaded March 18, 2016) for functions of a representative protein from each of the remaining 319 orthologous groups. These annotations were then evaluated for functions required for psilocybin synthesis and secretion. Candidate PS genes were defined as being present in all PS^+^ genomes and three to four genomes total. Clustering of all candidate genes was simultaneously evaluated in a database of 618 fungal proteomes by identifying overlapping pairs of query gene homologs (as retrieved by usearch version 8.0.1517 [Edgar 2010] using the ublast algorithm and retaining sequences with at least 45% amino acid similarity) that were separated by no more than six intervening genes in each genome. Cluster boundaries were defined by repeating the cluster search using a window of up to 10 genes on either side of each cluster locus, and identifying convergence of synteny and gene phylogeny consistent with PS distribution.

#### Conservation of local synteny

Proteins from each PS cluster locus were used to query the local proteome database for homologs using usearch version 8.0.1517 with an e‐value cutoff of 1 × 10^−5^ and minimum amino acid similarity of 0.4. Regions of shared synteny were retrieved by identifying overlapping pairs of query gene homologs that were separated by no more than six intervening genes in each genome. Orthology/xenology of genes in regions of putatively shared synteny was manually verified using phylogenetic trees (see Phylogenetic Analyses).

#### Detecting genes unique to dung‐ and wood‐associated fungi

Using the set of 32,326 Agaricales gene homology groups (AGHs) derived from the phylogenomic analysis of the Agaricales (see *Phylogenomic analyses of Agaricales* next), we identified genes restricted to dung‐associated fungi by selecting AGHs present in three or more dung‐associated fungal species (*Agrocybe pediades, Pa. cyanescens, Bolbitius vitellinus, Coprinopsis cinerea, Cyathus striatus, Crucibulum laeve*) that were absent among nondung‐associated wood decay (*Gy. dilepis, Gy. chrysopellus, Ga. marginata, Ps. cyanescens, Pholiota conissans, H. sublateritium, Crepidotus variabilis, Pluteus cervinus, Fibulorhizoctonia sp*.) and mycorrhizal (*Hebeloma cylindrosporum, Laccaria bicolor, Cortinarius glaucopus, Piloderma croceum*) fungal species. We similarly identified genes restricted to wood‐associated fungi or ectomycorrhizal fungi, with the added restriction that the AGHs be present in four or more wood‐associated fungi or present in two or more ectomycorrhizal fungi, respectively. We chose minimum cutoffs of 3, 4, and 2 for identifying dung‐, wood‐, and ectomycorrhizal‐specific genes, respectively, to avoid selecting AGHs whose apparent association with ecological lifestyle is confounded by phylogenetic relatedness (Maddison and FitzJohn 2015). These cutoffs ensured that, based on our sample of Agaricales species, the distribution of any given AGH found in fungi with an ecology of interest was polyphyletic (Fig. 3A). *Cyathus striatus and Crucibulum laeve* were considered to be both dung and wood associated.

#### Ordination of Agaricales genome content

Again using the set of 32,326 AGHs (see 2.4.1 *Phylogenomic analyses of Agaricales* next), we first discarded AGHs present in only one genome as well as those present in all 16 Agaricales genomes. For each Agaricales genome, we then counted the number of proteins assigned to each of the 10,998 remaining AGHs. The genome × AGH count matrix was then subjected to principal component analysis (PCA) in R using the default “prcomp” function, setting the AGH variables to be zero centered and to have unit variance (R Core Team 2015). Results were visualized using the R packages “ggplot2” and “ggrepel” (Wickham 2009; Slowikowski 2016). We then selected the AGH variables with high positive or low negative loading scores for the first two PCs. The minimum cutoff for what constituted a high (for positive scores) or low (for negative score) score for a given PC was set to the 95th percentile of all positive or all negative scores, accordingly. Tests for COG category enrichment among the proteins assigned to selected AGHs compared to the background of all functionally annotated proteins in the set of 10,998 AGHs were performed using a one‐tailed Fisher's exact test (*P* < 0.05), with Bonferroni correction for testing of multiple sets of AGHs (those contributing to positive PC1, positive PC2, negative PC1, or negative PC2).

### PHYLOGENETIC ANALYSES

#### Phylogenomic analyses of Agaricales

To generate a species tree, 16 Agaricales proteomes along with *Pi. croceum* and *Fibulorhizoctonia* sp. were clustered using OrthoMCL version 1.4 with an inflation value of 2.0, resulting in 32,326 Agaricales gene homology groups (AGHs). AGHs were then sampled randomly, tested for 1:1 sequence:species relationships, and then subjected to automated phylogenetic analysis. Sequences were aligned with mafft version 7.221 (Katoh and Standley 2013) using default parameters, and ambiguously aligned characters were removed from the resulting alignment with TrimAl version 1.4 (Capella‐Gutiérrez et al. 2009) using the automated1 algorithm. The best model of protein evolution was determined using prottest version 3.4 (Darriba et al. 2011) according to the Akaike information criteria (AICC). Phylogenetic analysis was performed in RAxML version 8.2.9 (Stamatakis 2014) mapping percentage of 100 rapid bootstraps to the best‐scoring ML tree. Trees were generated until 100 gene trees with greater than 70% average bootstrap support were obtained. The majority rule extended consensus of the 100 trees was computed in RAxML and used to constrain the final maximum likelihood analysis of the second largest subunit of RNA polymerase II (RPB2) as above, to represent the species tree. Internode certainty of the species tree was computed using all bootstraps from all genes, and tree certainty was computed using all maximum likelihood trees in RAxML.

#### Degenerate PCR screening for PsiD and PsiK gene homologs

Degenerate PCR was performed by using degenerate primers (Supporting Information Data) and genomic DNA extracted from: *Ps. azurescens, Ps. aeruginascens*, *Ps. cubensis*, and *C. smithii* as templates. The degenerate oligonucleotide primers were designed based on the nucleotide alignments of respective genes from the sequenced genomes of: *Ps. cyanescens*, *Gy. dilepis*, and *Pa. cyanescens* described in this study. Briefly, 100–300 ng of genomic DNA was used as template with 1 μM each of oligonucleotide primer pairs using Platinum Taq DNA polymerase (#11304‐011, Invitrogen, Waltham, MA, USA) following manufacturer's instructions. The PCR‐amplified DNA fragments were analyzed by agarose gel electrophoresis, gel purified using QIAEX II gel extraction kit (#20021, Qiagen, Germantown, MA, USA) and finally each purified PCR DNA fragment(s) sequence was determined by dye‐terminator sequencing. Amplicon sequences are reported by manually determined consensus of forward and reverse primer extensions.

#### Analysis of PS genes

To generate PS gene trees, homologs of PS protein sequences were identified in a 618 proteome database using usearch with an e‐value cutoff of 1 × 10^−5^ and minimum amino acid similarity of 0.4. Sequences were aligned with mafft using default parameters, and ambiguously aligned characters were removed from the resulting alignment with TrimAl using the automated1 algorithm. A preliminary phylogenetic analysis was performed using fasttree version 2.1 (Price et al. 2010). The original set of sequences was then manually reduced to a strongly supported clade of less than 250 sequences, which included the query sequences and was supported by >0.90 local support, followed by realignment and removal of characters according to the above parameters. The best model of protein evolution was determined using prottest according to the AICC. Phylogenetic analysis was performed in RAxML mapping percentage of 100 rapid bootstraps to the best‐scoring ML tree.

HGT hypothesis testing of select genes was performed by comparing the site log‐likelihood scores of the optimal and vertical inheritance‐constrained topologies (Supporting Information Data). The null hypothesis of vertical inheritance was rejected if the log‐likelihood was significantly worse (*p* < 0.05) using the Approximately Unbiased Test as implemented in Consel version 0.20 (Shimodaira and Hasegawa 2001). Gene tree–species tree reconciliation was conducted in Notung version 2.9 (Alderson et al. 2017) using the phylogenomic tree (above) for the species tree and the best ML tree as the gene tree. The DTL (Duplication Transfer and Loss allowed) model costs were assigned as 1.5, 3.0, and 1.0, respectively, and the DL model costs were assigned as 1.5 and 1.0, respectively. The edge weight threshold was set at 1.0.

#### Genome scale analyses of HGT

##### Detecting horizontal transfers from *Psilocybe* lineage to *Pa. cyanescens*

We developed a targeted bioinformatic pipeline drawing upon established methods for testing hypotheses of HGT to detect genes with signatures of HGT from the *Psilocybe* lineage to the *Pa. cyanescens* genome (Fig. S6). Given the large number of species included in our database and our interest in testing hypotheses of HGT between two specific lineages, we chose not to use existing methods that model duplication and loss events in addition to HGT over the entire query tree, as they would have been unnecessarily computationally intensive to use on a genome‐wide basis with large gene trees (Szöllősi et al. 2013). Briefly, for the first filtering step, we used the entire *Pa. cyanescens* proteome to query our local proteome database with DIAMOND BLASTp (‐sensitive and e‐value = 1 × 10^−4^ (Butchfink et al. 2015). We retained *Pa. cyanescens* queries that had a *Psilocybe* sequence as their best‐scoring hit, and then used these queries to conduct a more refined search of the local proteome database using BLASTp (e‐value = 1 × 10^−4^) (Altschul et al. 1990). For the second filtering step, preliminary phylogenetic trees were built for each query and its associated hits using fasttree version 2.1, as above, and rooted at the sequence most distant from the query. Trees in which *Pa. cyanescens* query sequences shared an immediate common ancestor with a *Psilocybe* sequence, and were separated by two or more ancestral nodes from other Agaricales and Atheliales sequences were retained. Large trees with more than 250 sequences were manually reduced to a strongly supported clade (>70% bootstrap support) containing at most 250 sequences and the *Pa. cyanescens* query. For the third filtering step, the sequences within this clade were then aligned, trimmed and analyzed in RAxML to construct a maximum likelihood tree with 100 rapid bootstraps, as above. Trees in which *Pa. cyanescens* queries shared an immediate common ancestor with *Psilocybe* and were nested within a larger clade of Hymenogastraceae and Strophariaceae sequences with more than two nodes of >70% bootstrap support were retained to be used in subsequent constraint analyses to test hypotheses of HGT, although no trees ended up meeting this criteria.

##### Detecting transfers of dung specific genes to *Pa. cyanescens*

*Panaeolus cyanescens* sequences in the set of homology groups unique to dung‐associated Agaricales (see above) were used as queries to search the local proteome database using BLASTp (e‐value = 1 × 10^−4^). All queries and their respective hits were assessed for evidence of HGT between dung‐associated fungi and *Pa. cyanescens* using a modified iteration of the HGT bioinformatic pipeline described above (see detecting horizontal transfers from *Psilocybe* lineage to *Pa. cyanescens*). Namely, for the second filtering step, trees containing *Pa. cyanescens* queries sharing an immediate ancestor with a dung‐associated Agaricales and separated by two or more ancestral nodes from nondung‐associated Agaricales species were retained. For the third filtering step, *Pa. cyanescens* queries sharing an immediate common ancestor with a dung‐associated Agaricales species and nested within a larger clade of nondung‐associated Agaricales species with more than two nodes of >70% bootstrap support were used in subsequent constraint analyses. We developed three null constraint scenarios of vertical inheritance to test for the likelihood of different HGT events throughout the tree of Pancy2_7201, the single sequence that was retained throughout all of the above filtering steps (Supporting Information Data 4). The site log likelihood scores of the optimal and constrained topologies were compared using the Approximately Unbiased Test as implemented in Consel version 0.20 (Shimodaira and Hasegawa 2001), and constrained hypotheses of vertical inheritance were rejected if their log likelihood was significantly worse (*P* < 0.05) than the optimal tree.

### ANALYSES OF ENZYME FUNCTION

#### Expression and purification of recombinant proteins

An oligo‐dT‐primed cDNA library was used as the template for obtaining the coding sequences of PsiD, and PsiK, by PCR amplification using Platinum Taq DNA polymerase (#11304‐011, Invitrogen, Waltham, MA, USA) and specially designed oligonucleotide primer pairs for enzyme‐free cloning into pETite C‐His Kan Vector (#49002‐1, Expresso T7 Cloning Kit, Lucigen Corp., Middleton, WI, USA) for C‐terminal His tag protein expression and purification. The oligonucleotide primer pairs used in the present study are listed in Supporting Information Data. The resulting recombinant plasmids—pETite‐PsiD and pETite‐PsiK—were transformed into the HI‐Control 10G host strain and following sequence verification freshly transformed into HI‐Control BL21(DE3) chemically competent cells for expression and purification, respectively, following manufacturer's instructions. Sequence verified transformant(s) were grown at 37°C until cultures reached an OD600 of 0.4–0.6, protein expressions were induced with 1 mM IPTG (isopropyl–d‐thiogalactopyranoside) (#I3301, Teknova, Hollister, CA, USA) for 4 h at 22–25°C, and cell lysates were prepared by using 1 mg/mL lysozyme (#3L2510, Teknova, Hollister, CA, USA) with incubation on ice for 30 min. Purification of 6xHis tagged recombinant proteins from cell lysates was performed under native conditions by affinity chromatography using His‐Pur Ni‐NTA superflow Agarose (#25215, Thermo Scientific, Waltham, MA, USA) loaded onto Poly‐Prep chromatography columns (#731‐1550, Bio‐Rad, Hercules, CA, USA). The purity of the eluted protein was analyzed by SDS‐PAGE, and protein concentrations were determined by using the Pierce BCA protein assay kit (#23227, Thermo Scientific, Waltham, MA, USA).

#### In vitro characterization of recombinant protein activity

All reactions were carried out in duplicates, and in two independent experiments.

##### Decarboxylase assay

Briefly, individual reactions were carried out in 1.5 mL Eppendorf vials using 600 μL of enzymatic reaction buffer containing; 80 mM Tris buffer (pH 7.5), 5mM MgCl_2_, 100 μM EDTA, ≤10 μg purified protein and 5 mM substrate concentrations. Reactions were incubated at 37°C for 20 h followed by freezing of the enzymatic reactions at −80°C. Finally, reactions were lyophilized, dissolved in 200 μL of pure HPLC grade methanol (A452‐4, Fisher Scientific, Columbus, OH, USA) and 50 μL and 1 μL aliquots of reaction mixtures were analyzed by the Varian 500‐MS ion trap mass spectrometer and by Waters ACQUITY® UPLC‐MS/MS TQD triple quadrupole mass spectrometer, respectively.

##### Phosphotransferase assay

Individual reactions were carried out in 1.5 mL Eppendorf vials using 100 μL of enzymatic reaction buffer containing; 50 mM Tris buffer (pH 8.0) and/or phosphate buffer, 50 mM KCl, 10 mM MgCl_2_, 1 mM DTT, 100 μM ATP/GTP, with/without 0.4 mM NADPH, ≤10μg purified protein, and 5 mM substrate concentrations. Reactions were incubated at 25°C for 30 min followed by a further incubation for 30 min at 30°C. The reactions were terminated by the addition of equal volume of cold HPLC grade methanol (A452‐4, Fisher Scientific, Columbus, OH, USA) and incubated at 4°C for 30 min. Finally, reactions were centrifuged at 16,000 × *g* for 10 min and aliquots (50 μL) of the resulting supernatants were directly used for Varian 500‐MS ion trap mass spectrometer measurements.

##### Mass spectrometric analysis of in vitro protein assays

The HPLC system consisted of the binary gradient LC/MS chromatography pump 212‐LC (Agilent, Santa Clara, CA, USA Technologies). The column was Polaris 3 C18‐A (4.6 < 150 mm, 5 μm; Agilent Technologies) (Waters Corp., Milford, MA) with a column temperature of 35–40°C. The mobile phase consisted of 0.1% acetic acid (A, aqueous) and acetonitrile (B) with a flow rate of 0.3 mL/min throughout. The gradient used for *decarboxylase assay* was as follows: 0–3 min of 5% B, 3–4 min of 5–10% B, 4–6 min of 10% B, 6–36 min of 10–30% B, 36–46 min of 100% B, and 46–50 min of 5% B again (adapted from Kalb et al. 2016). For UPLC‐MS/MS measurements, the column was Cortecs UPLC C18 (2.1 < 100 mm. 1.6 μm, #186007095) (Waters Corp., Milford, MA) with a column temperature of 50°C. The mobile phase consisted of 0.1% acetic acid (A, aqueous) and acetonitrile (B) with a flow rate of 0.3 mL/min throughout. The gradient used was as follows: 0–0.3 min of 5% B, 0.3–4 min of 5–10% B, 4–6 min of 10% B, 6–8 min of 10–50% B, 8–12 min of 50–100% B, and 12–15 min of 5% B again. The gradient used for *phosphotransferase assay* was as follows: 0–18 min of 5–55% B, 18–32 min of 55–100% B, 32–35 min of 100% B, and 35–40 min of 5% B again (adapted from Manevski et al. 2009).

Mass spectrometry data were acquired using electrospray ionization source of Agilent's Varian, Inc. 500‐MS ion trap instrument under positive ion mode. Nitrogen gas was used as nebulizer (35–40 psi) and helium gas in the ion trap. The LC/MS/MS analyses were carried out using the TurboDDS‐enhanced scan mode with a scan range of 50–2000 *m*/*z* (*decarboxylase assay*) and 50–750 *m*/*z* (*phosphotransferase assay*) with a capillary voltage of 80 V and RF loading of 100%. MS data acquisition and analysis was performed using the Varian MS workstation Version 6.9. Mass spectrometric data using Waters ACQUITY® TQD was acquired using electrospray ionization source of Waters TQ Detector under positive ion mode. Nitrogen was used as API source gas and helium was used as the collision gas. The tune and MRM parameters for each substrate and product ion type (tryptophan, 5HTP, tyrosine, phenylalanine, tryptamine, and tyramine) were generated using the IntelliStart Technology.

Associate Editor: L. Bromham

## Supporting information

Supplemental Data

**Figure S1. (**A) Mass chromatogram of LC‐MS/MS.
**Figure S2. (**A–E) RAxML phylogenies of psilocybin (PS) genes with support shown out of 100 bootstraps.
**Figure S3**. Gene tree–species tree reconciliation in NOTUNG.
**Figure S4**. Schematics of the bioinformatic pipelines used to detect horizontal gene transfers (HGTs) to *Panaeolus cyanescens*, along with the number of sequences retained at each step.
