# Supplementary material for: Horizontal gene cluster transfer increased hallucinogenic mushroom diversity
Source: Evol Lett. 2018 Feb 27;2(2):88–101. doi: 10.1002/evl3.42 (PMC6121855; doi:10.1002/evl3.42)
Supplement: Supplementary file 2 — Figure S1. (A) Mass chromatogram of LC‐MS/MS. Figure S2. (A–E) RAxML phylogenies of psilocybin (PS) genes with support shown out of 100 bootstraps. Figure S3. Gene tree–species tree reconciliation in NOTUNG. Figure S4. Schematics of the bioinformatic pipelines used to detect horizontal gene transfers (HGTs) to Panaeolus cyanescens, along with the number of sequences retained at each step. [file EVL3-2-88-s001.pdf]

Horizontal gene cluster transfer increased hallucinogenic mushroom diversity

Supplementary Figure Legends:

Figure S1. A. Mass Chromatogram of LC-MS/MS. Multiple-Reaction-Monitoring (MRM) chromatogram of tryptophan reaction (a). MRM-chromatogram of mass-to-charge ratio (m/z) 205 [M+H]+ (peak corresponding to tryptophan mass) and its MS/MS looking for one of its daughter ions at m/z 146 (b). MRM chromatogram of tryptophan+tryptophan decarboxylase (PsiD) reaction, showing additional peaks at retention time (tR) of 3.38, 4.48 and 5.09 (c). MRM-chromatogram of m/z 144.1 [M+H]+ (characteristic tryptamine peak mass) and its MS/MS looking for one of its daughter ions at m/z 117 (d).

Figure S2. A-E) RAxML phylogenies of psilocybin (PS) genes with support shown out of 100 bootstraps. Terminals indicate JGI accession numbers for protein models. Clustered PS genes are indicated in blue. A. tryptophan decarboxylase (PsiD); B. N-methyltransferase (PsiM); C. hydroxylase (PsiH); D. phosphotransferase (PsiK); E. transporter (PsiT). F) Phylogeny of ferric reductase, where sequences putatively involved in a horizontal gene transfer event are indicated in red.

Figure S3. Gene tree-species tree reconciliation in NOTUNG. D=duplication, T=horizontal transfer, L=loss of hypothetical genes.

Figure S4. Schematics of the bioinformatic pipelines used to detect horizontal gene transfers (HGTs) to *Panaeolus cyanescens*, along with the number of sequences retained at each step. A) Schematic describing the pipeline for the identification of HGT from *Psilocybe cyanescens* to *Pa. cyanescens*. B) Schematic describing the pipeline for the identification of HGT from dung-associated Agaricales to *Pa. cyanescens*.

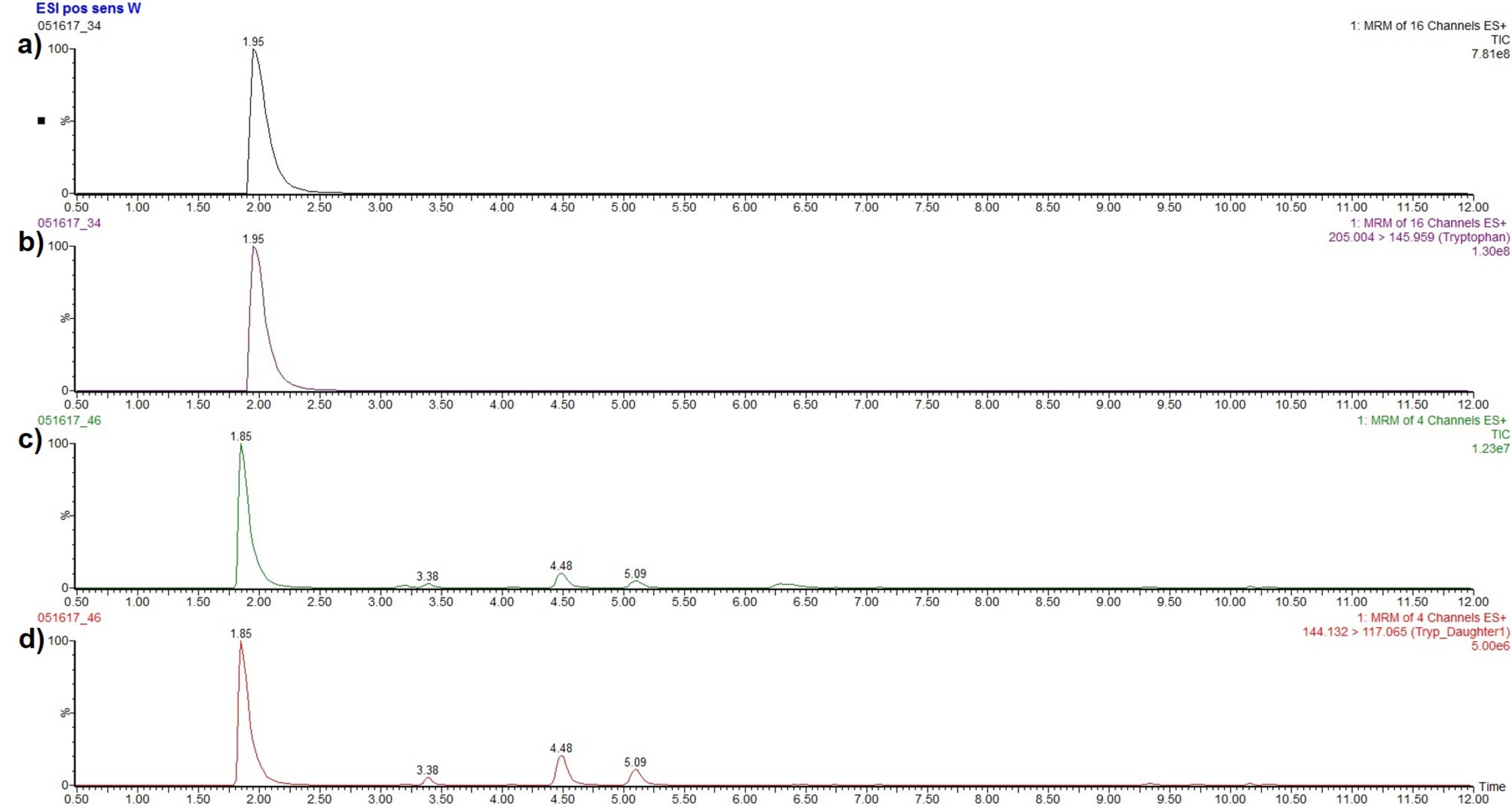
ESI pos sens W
051617_34
1: MRM of 16 Channels ES+
TIC
7.81e8
a)
1.95
051617_34
1: MRM of 16 Channels ES+
205.004 > 145.959 (Tryptophan)
1.30e8
b)
1.95
051617_46
1: MRM of 4 Channels ES+
TIC
1.23e7
c)
1.85
3.38
4.48
5.09
051617_46
1: MRM of 4 Channels ES+
144.132 > 117.065 (Tryp_Daughter1)
5.00e6
d)
1.85
3.38
4.48
5.09
Time

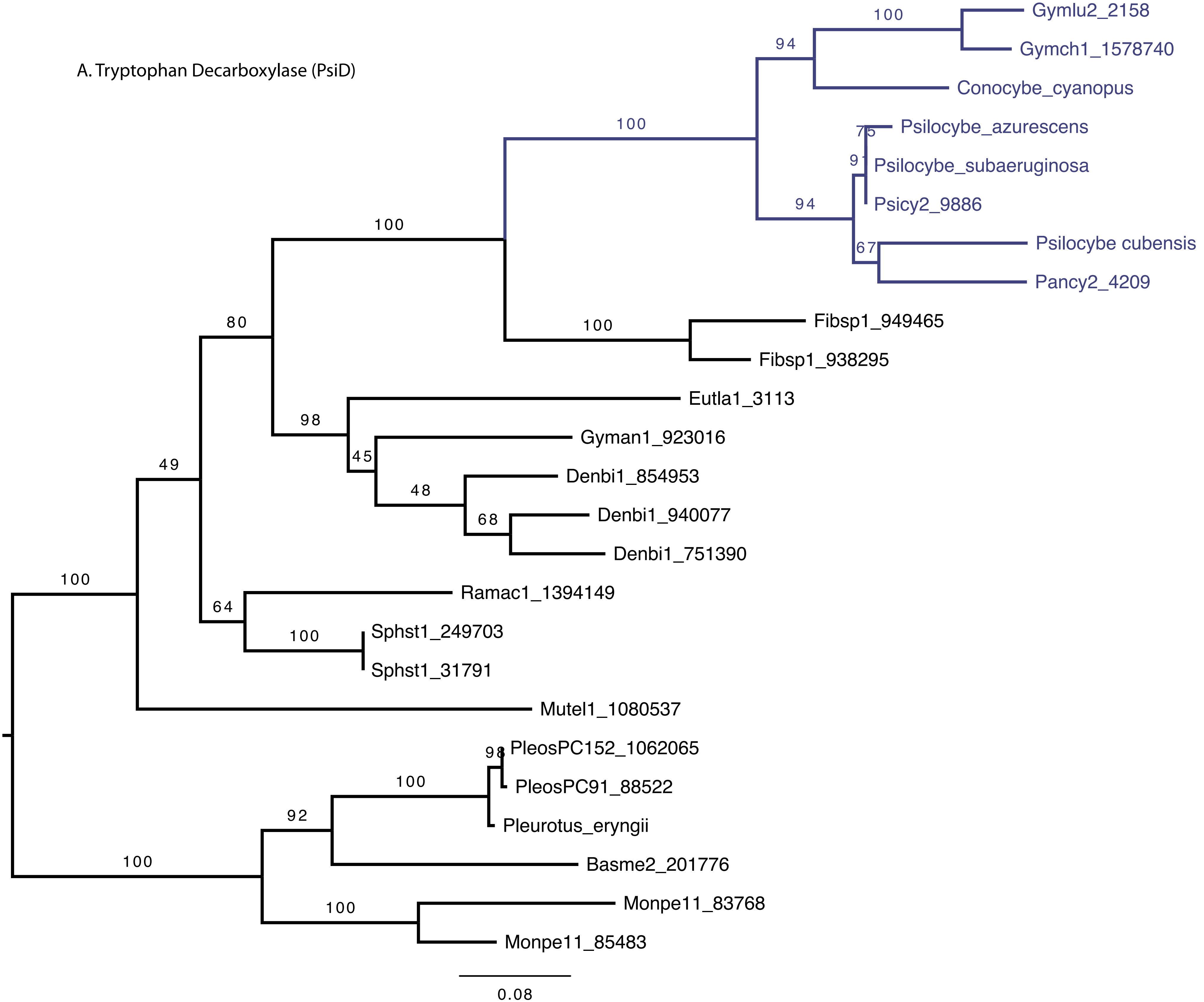
A. Tryptophan Decarboxylase (PsiD)
Gymlu2_2158
Gymch1_1578740
Conocybe_cyanopus
Psilocybe_azurescens
Psilocybe_subaeruginosa
Psicy2_9886
Psilocybe cubensis
Pancy2_4209
Fibsp1_949465
Fibsp1_938295
Eutla1_3113
Gyman1_923016
Denbi1_854953
Denbi1_940077
Denbi1_751390
Ramac1_1394149
Sphst1_249703
Sphst1_31791
Mutel1_1080537
PleosPC152_1062065
PleosPC91_88522
Pleurotus_eryngii
Basme2_201776
Monpe11_83768
Monpe11_85483
0.08

B. N-methyltransferase (PsiM)

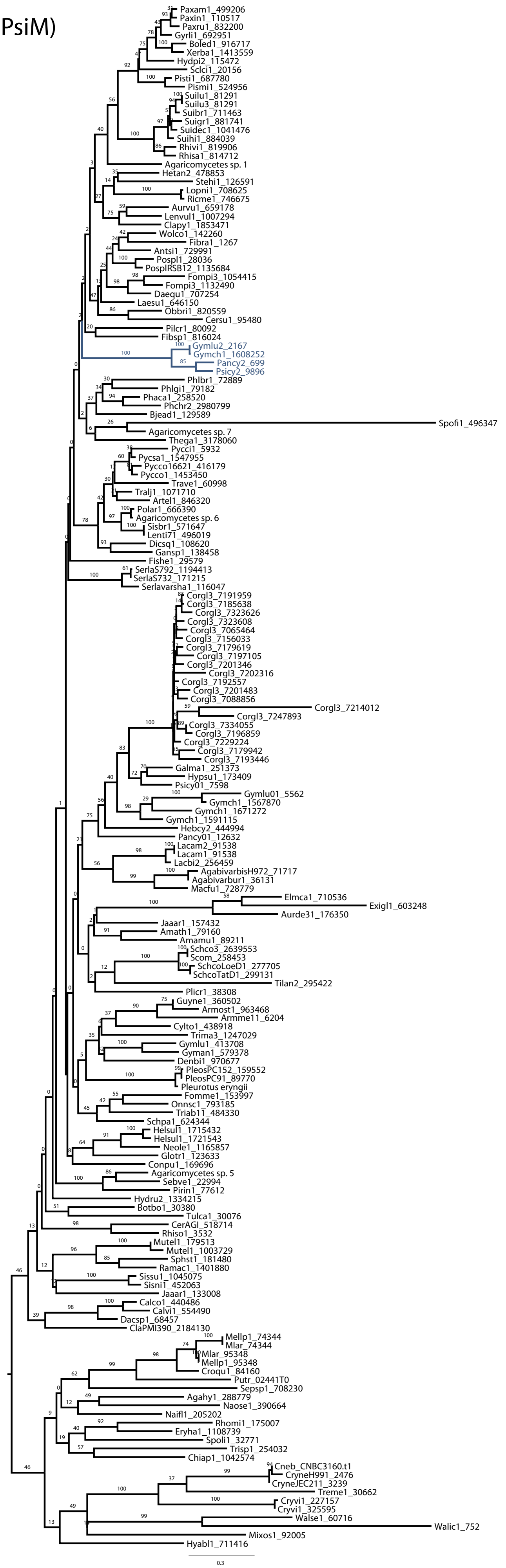

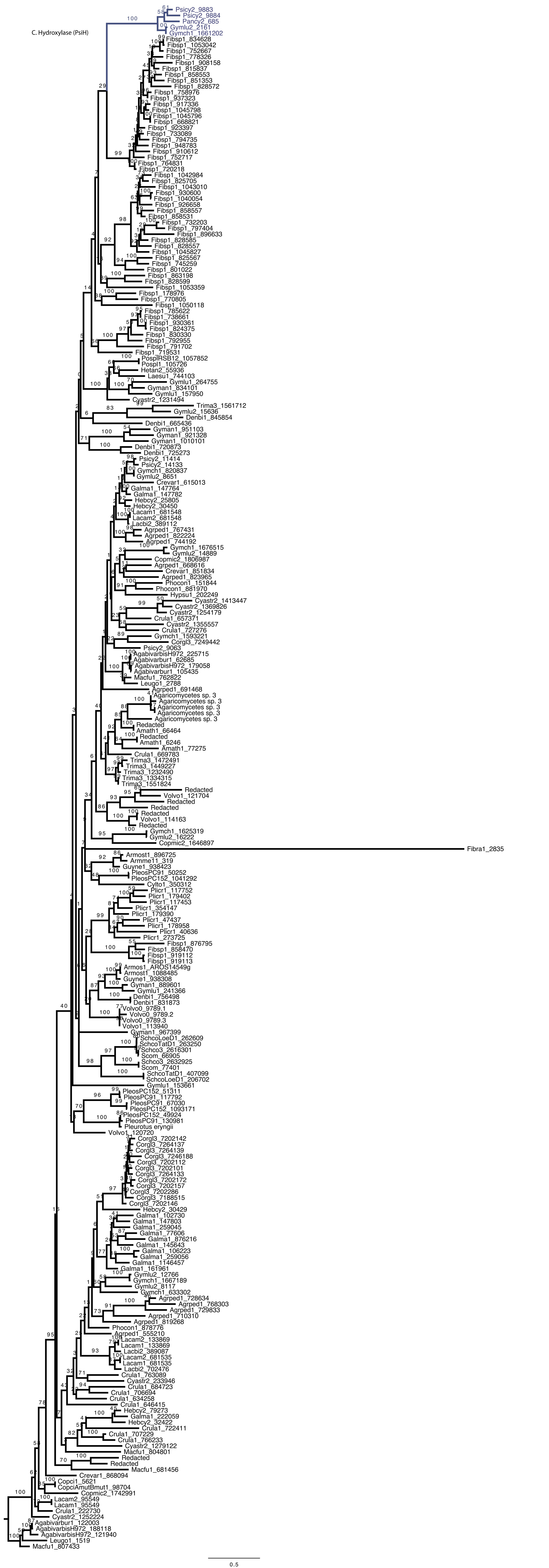
C. Hydroxylase (PsiH)
Psicy2_9883
Psicy2_9884
Pancy2_685
Gymlu2_2161
Gymch1_1661202
Fibsp1_834628
Fibsp1_1053042
Fibsp1_752667
Fibsp1_778326
Fibsp1_908158
Fibsp1_815837
Fibsp1_858553
Fibsp1_851353
Fibsp1_828572
Fibsp1_758976
Fibsp1_937323
Fibsp1_917336
Fibsp1_1045798
Fibsp1_1045796
Fibsp1_668821
Fibsp1_923397
Fibsp1_733089
Fibsp1_794735
Fibsp1_948783
Fibsp1_910612
Fibsp1_752717
Fibsp1_764831
Fibsp1_720218
Fibsp1_1042984
Fibsp1_825705
Fibsp1_1043010
Fibsp1_930600
Fibsp1_1040054
Fibsp1_926658
Fibsp1_858557
Fibsp1_858531
Fibsp1_732203
Fibsp1_797404
Fibsp1_896633
Fibsp1_828585
Fibsp1_828557
Fibsp1_1045827
Fibsp1_825567
Fibsp1_745259
Fibsp1_801022
Fibsp1_863198
Fibsp1_828599
Fibsp1_1053359
Fibsp1_178976
Fibsp1_770805
Fibsp1_1050118
Fibsp1_785622
Fibsp1_738661
Fibsp1_930361
Fibsp1_824375
Fibsp1_830330
Fibsp1_792955
Fibsp1_791702
Fibsp1_719531
PosplRSB12_1057852
Pospl1_105726
Hetan2_55936
Laesu1_744103
Gymlu1_264755
Gyman1_834101
Gymlu1_157950
Cyastr2_1231494
Trima3_1561712
Gymlu2_15636
Denbi1_845854
Denbi1_665436
Gyman1_951103
Gyman1_921328
Gyman1_1010101
Denbi1_720873
Denbi1_725273
Psicy2_11414
Psicy2_14133
Gymch1_820837
Gymlu2_8651
Crevar1_615013
Galma1_147764
Galma1_147782
Hebcy2_25805
Hebcy2_30450
Lacam1_681548
Lacam2_681548
Lacbi2_389112
Agrped1_767431
Agrped1_822224
Agrped1_744192
Gymch1_1676515
Gymlu2_14889
Copmic2_1806987
Agrped1_668616
Crevar1_851834
Agrped1_823965
Phocon1_151844
Phocon1_881970
Hypsu1_202249
Cyastr2_1413447
Cyastr2_1369826
Cyastr2_1254179
Crula1_657371
Cyastr2_1355557
Crula1_727276
Gymch1_1593221
Corgl3_7249442
Psicy2_9063
AgabivarbisH972_225715
Agabivarbur1_62685
AgabivarbisH972_179058
Agabivarbur1_105435
Macfu1_762822
Leugo1_2788
Agrped1_691468
Agaricomycetes sp. 3
Agaricomycetes sp. 3
Agaricomycetes sp. 3
Agaricomycetes sp. 3
Agaricomycetes sp. 3
Redacted
Amath1_66464
Redacted
Amath1_6246
Amath1_77275
Crula1_669783
Trima3_1472491
Trima3_1449227
Trima3_1232490
Trima3_1334315
Trima3_1551824
Redacted
Volvo1_121704
Redacted
Redacted
Redacted
Volvo1_114163
Redacted
Gymch1_1625319
Gymlu2_16222
Copmic2_1646897
Fibra1_2835
Armost1_896725
Armme11_319
Guyne1_938423
PleosPC91_50252
PleosPC152_1041292
Cylto1_350312
Plicr1_117752
Plicr1_179402
Plicr1_117453
Plicr1_354147
Plicr1_179390
Plicr1_47437
Plicr1_178958
Plicr1_40636
Plicr1_273725
Fibsp1_876795
Fibsp1_858470
Fibsp1_919112
Fibsp1_919113
Armos1_AROS14549g
Armost1_1088485
Guyne1_938308
Gyman1_889601
Gymlu1_241366
Denbi1_756498
Denbi1_831873
Volvo0_9789.1
Volvo0_9789.2
Volvo0_9789.3
Volvo1_113940
Gyman1_967399
SchcoLoeD1_262609
SchcoTatD1_263250
Schco3_2616301
Scom_66905
Schco3_2632925
Scom_77401
SchcoTatD1_407099
SchcoLoeD1_206702
Gymlu1_153661
PleosPC152_51311
PleosPC91_117792
PleosPC91_67030
PleosPC152_1093171
PleosPC152_49924
PleosPC91_130981
Pleurotus eryngii
Volvo1_120720
Corgl3_7202142
Corgl3_7264137
Corgl3_7264139
Corgl3_7246188
Corgl3_7202112
Corgl3_7202101
Corgl3_7264133
Corgl3_7202172
Corgl3_7202157
Corgl3_7202286
Corgl3_7188515
Corgl3_7202146
Hebcy2_30429
Galma1_102730
Galma1_147803
Galma1_259045
Galma1_77606
Galma1_876216
Galma1_145643
Galma1_106223
Galma1_259056
Galma1_1146457
Galma1_161961
Gymlu2_12766
Gymch1_1667189
Gymlu2_8117
Gymch1_633302
Agrped1_728634
Agrped1_768303
Agrped1_729833
Agrped1_710310
Agrped1_819268
Phocon1_878776
Agrped1_555210
Lacam2_133869
Lacam1_133869
Lacbi2_389087
Lacam2_681535
Lacam1_681535
Lacbi2_702476
Crula1_763089
Cyastr2_233946
Crula1_684723
Crula1_706694
Crula1_634258
Crula1_646415
Hebcy2_79273
Galma1_222059
Hebcy2_32422
Crula1_722411
Crula1_707229
Crula1_766233
Cyastr2_1279122
Macfu1_804801
Redacted
Redacted
Macfu1_681456
Crevar1_868094
Copci1_5621
CopciAmutBmut1_98704
Copmic2_1742991
Lacam2_95549
Lacam1_95549
Crula1_222730
Cyastr2_1252224
Agabivarbur1_122003
AgabivarbisH972_188118
AgabivarbisH972_121940
Leugo1_1519
Macfu1_807433
0.5

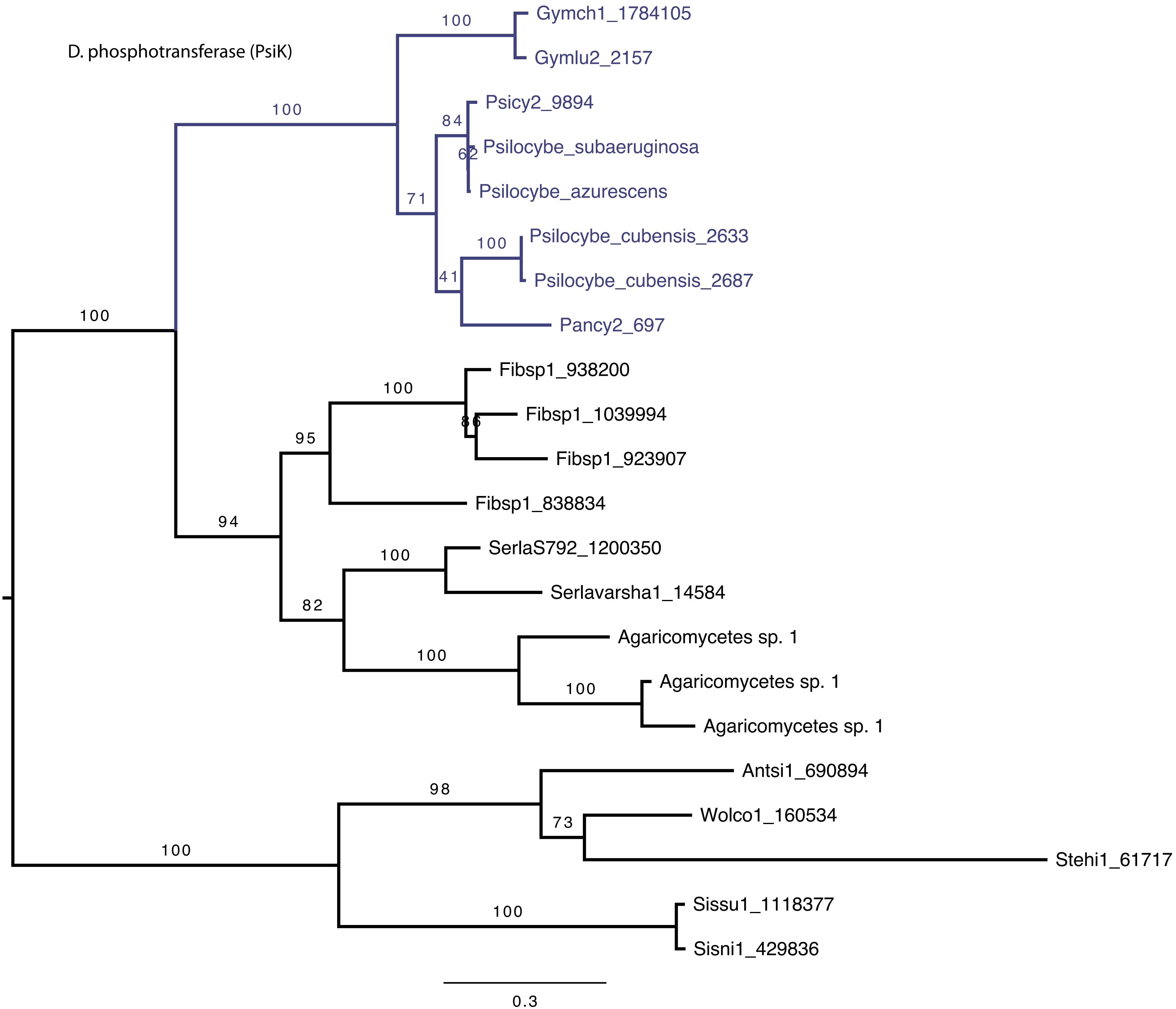

D. phosphotransferase (PsiK)
Gymch1_1784105
Gymlu2_2157
Psicy2_9894
Psilocybe_subaeruginosa
Psilocybe_azurescens
Psilocybe_cubensis_2633
Psilocybe_cubensis_2687
Pancy2_697
Fibsp1_938200
Fibsp1_1039994
Fibsp1_923907
Fibsp1_838834
SerlaS792_1200350
Serlavarsha1_14584
Agaricomycetes sp. 1
Agaricomycetes sp. 1
Agaricomycetes sp. 1
Antsi1_690894
Wolco1_160534
Stehi1_61717
Sissu1_1118377
Sisni1_429836
100
100
84
62
71
100
41
100
100
86
95
94
100
82
100
100
98
73
100
100
0.3

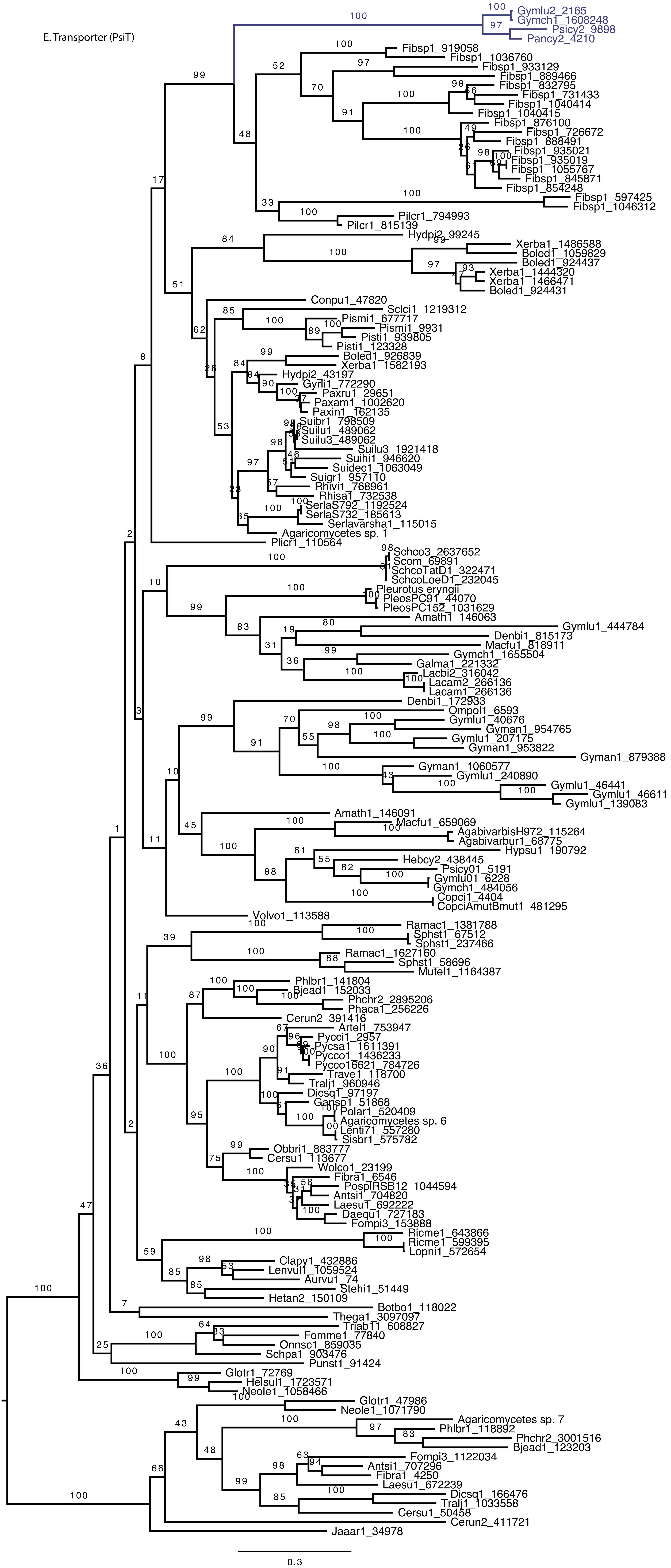
E. Transporter (PsiT)
Gymlu2_2165
Gymch1_1608248
Psicy2_9898
Pancy2_4210
Fibsp1_919058
Fibsp1_1036760
Fibsp1_933129
Fibsp1_889466
Fibsp1_832795
Fibsp1_731433
Fibsp1_1040414
Fibsp1_1040415
Fibsp1_876100
Fibsp1_726672
Fibsp1_888491
Fibsp1_935021
Fibsp1_935019
Fibsp1_1055767
Fibsp1_845871
Fibsp1_854248
Fibsp1_597425
Fibsp1_1046312
Pilcr1_794993
Pilcr1_815139
Hydpi2_99245
Xerba1_1486588
Boled1_1059829
Boled1_924437
Xerba1_1444320
Xerba1_1466471
Boled1_924431
Conpu1_47820
Sclci1_1219312
Pismi1_677717
Pismi1_9931
Pisti1_939805
Pisti1_123328
Boled1_926839
Xerba1_1582193
Hydpi2_43197
Gyrli1_772290
Paxru1_29651
Paxam1_1002620
Paxin1_162135
Suibr1_798509
Suilu1_489062
Suilu3_489062
Suilu3_1921418
Suihi1_946620
Suidec1_1063049
Suigr1_957110
Rhivi1_768961
Rhisa1_732538
SerlaS792_1192524
SerlaS732_185613
Serlavarsha1_115015
Agaricomycetes sp. 1
Plicr1_110564
Schco3_2637652
Scom_69891
SchcoTatD1_322471
SchcoLoeD1_232045
Pleurotus eryngii
PleosPC91_44070
PleosPC152_1031629
Amath1_146063
Gymlu1_444784
Denbi1_815173
Macfu1_818911
Gymch1_1655504
Galma1_221332
Lacbi2_316042
Lacam2_266136
Lacam1_266136
Denbi1_172933
Ompol1_6593
Gymlu1_40676
Gyman1_954765
Gymlu1_207175
Gyman1_953822
Gyman1_879388
Gyman1_1060577
Gymlu1_240890
Gymlu1_46441
Gymlu1_46611
Gymlu1_139083
Amath1_146091
Macfu1_659069
AgabivarbisH972_115264
Agabivarbur1_68775
Hypsu1_190792
Hebcy2_438445
Psicy01_5191
Gymlu01_6228
Gymch1_484056
Copci1_4404
CopciAmutBmut1_481295
Volvo1_113588
Ramac1_1381788
Sphst1_67512
Sphst1_237466
Ramac1_1627160
Sphst1_58696
Mutel1_1164387
Phlbr1_141804
Bjead1_152033
Phchr2_2895206
Phaca1_256226
Cerun2_391416
Artel1_753947
Pycci1_2957
Pycsa1_1611391
Pycco1_1436233
Pycco16621_784726
Trave1_118700
Tralj1_960946
Dicsq1_97197
Gansp1_51868
Polar1_520409
Agaricomycetes sp. 6
Lenti71_557280
Sisbr1_575782
Obbri1_883777
Cersu1_113677
Wolco1_23199
Fibra1_6546
PospIRSB12_1044594
Antsi1_704820
Laesu1_692222
Daequ1_727183
Fompi3_153888
Ricme1_643866
Ricme1_599395
Lopni1_572654
Clapy1_432886
Lenvul1_1059524
Aurvu1_74
Stehi1_51449
Hetan2_150109
Botbo1_118022
Thega1_3097097
Triab11_608827
Fomme1_77840
Onnsc1_859035
Schpa1_903476
Punst1_91424
Glotr1_72769
Helsul1_1723571
Neole1_1058466
Glotr1_47986
Neole1_1071790
Agaricomycetes sp. 7
Phlbr1_118892
Phchr2_3001516
Bjead1_123203
Fompi3_1122034
Antsi1_707296
Fibra1_4250
Laesu1_672239
Dicsq1_166476
Tralj1_1033558
Cersu1_50458
Cerun2_411721
Jaaar1_34978
0.3

F. ferric reductase

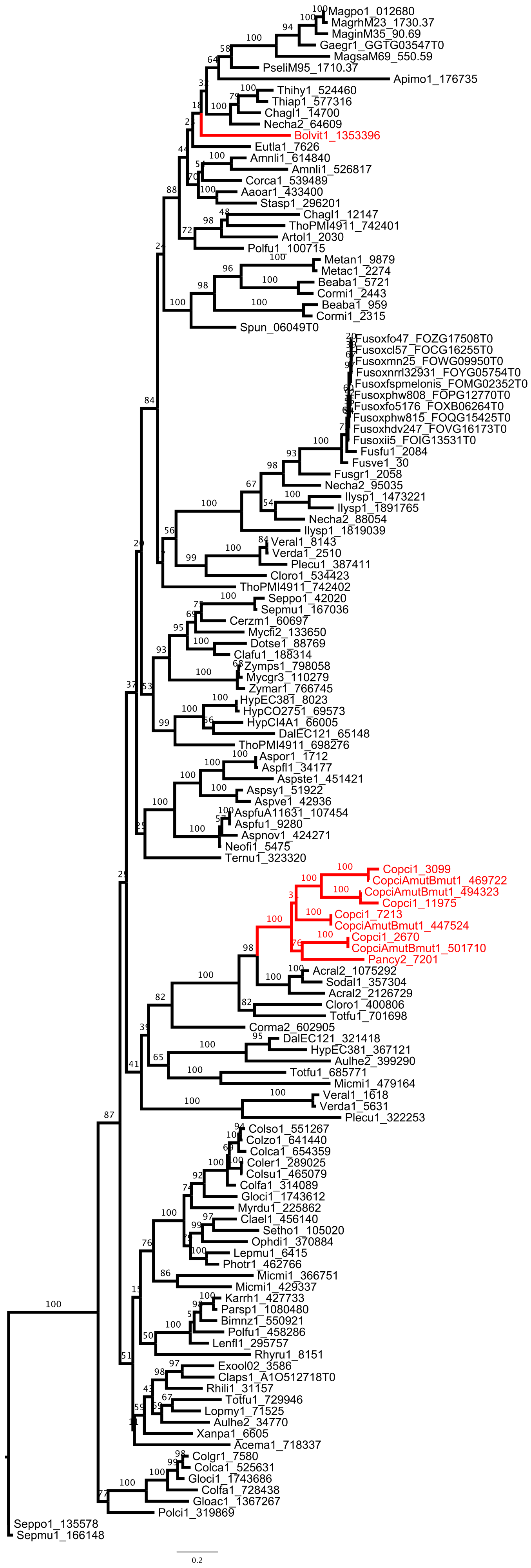

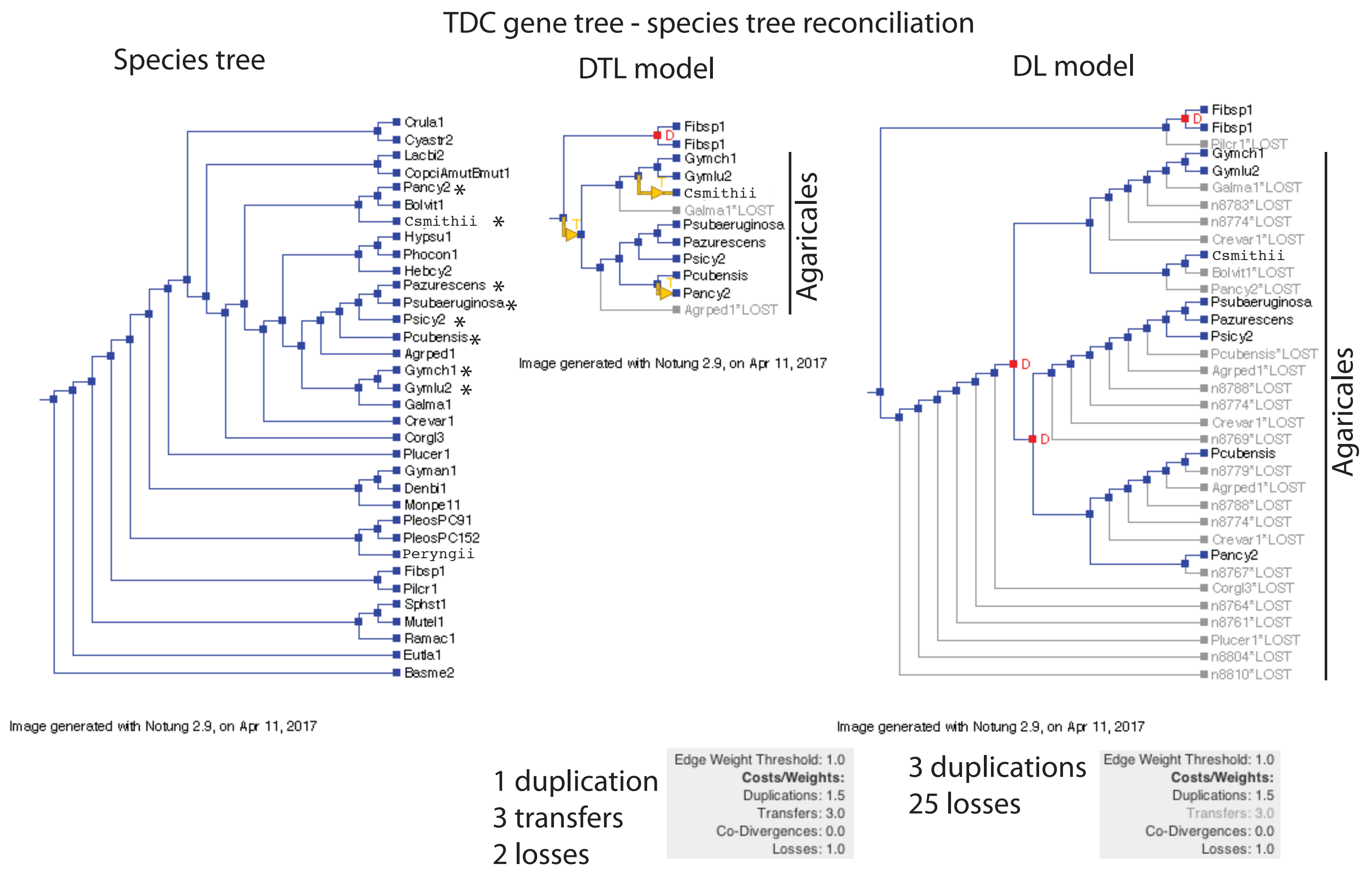

TDC gene tree - species tree reconciliation
Species tree
DTL model
DL model
Crula1
Cyastr2
Lacbi2
CopciAmutBmut1
Pancy2 *
Bolvit1
Csmithii *
Hypsu1
Phocon1
Hebcy2
Pazurescens *
Psubaeruginosa *
Psicy2 *
Pcubensis *
Agrped1
Gymch1 *
Gymlu2 *
Galma1
Crevar1
Corgl3
Plucer1
Gyman1
Denbi1
Monpe11
PleosPC91
PleosPC152
Peryngii
Fibsp1
Pilcr1
Sphst1
Mutel1
Ramac1
Eutla1
Basme2
Image generated with Notung 2.9, on Apr 11, 2017
Fibsp1
Fibsp1
Gymch1
Gymlu2
Csmithii
Galma1*LOST
Psubaeruginosa
Pazurescens
Psicy2
Pcubensis
Pancy2
Agrped1*LOST
Agaricales
Image generated with Notung 2.9, on Apr 11, 2017
Fibsp1
Fibsp1
Pilcr1*LOST
Gymch1
Gymlu2
Galma1*LOST
n8783*LOST
n8774*LOST
Crevar1*LOST
Csmithii
Bolvit1*LOST
Pancy2*LOST
Psubaeruginosa
Pazurescens
Psicy2
Pcubensis*LOST
Agrped1*LOST
n8788*LOST
n8774*LOST
Crevar1*LOST
n8769*LOST
Pcubensis
n8779*LOST
Agrped1*LOST
n8788*LOST
n8774*LOST
Crevar1*LOST
Pancy2
n8767*LOST
Corgl3*LOST
n8764*LOST
n8761*LOST
Plucer1*LOST
n8804*LOST
n8810*LOST
Agaricales
Image generated with Notung 2.9, on Apr 11, 2017
1 duplication
3 transfers
2 losses
Edge Weight Threshold: 1.0
Costs/Weights:
Duplications: 1.5
Transfers: 3.0
Co-Divergences: 0.0
Losses: 1.0
3 duplications
25 losses
Edge Weight Threshold: 1.0
Costs/Weights:
Duplications: 1.5
Transfers: 3.0
Co-Divergences: 0.0
Losses: 1.0

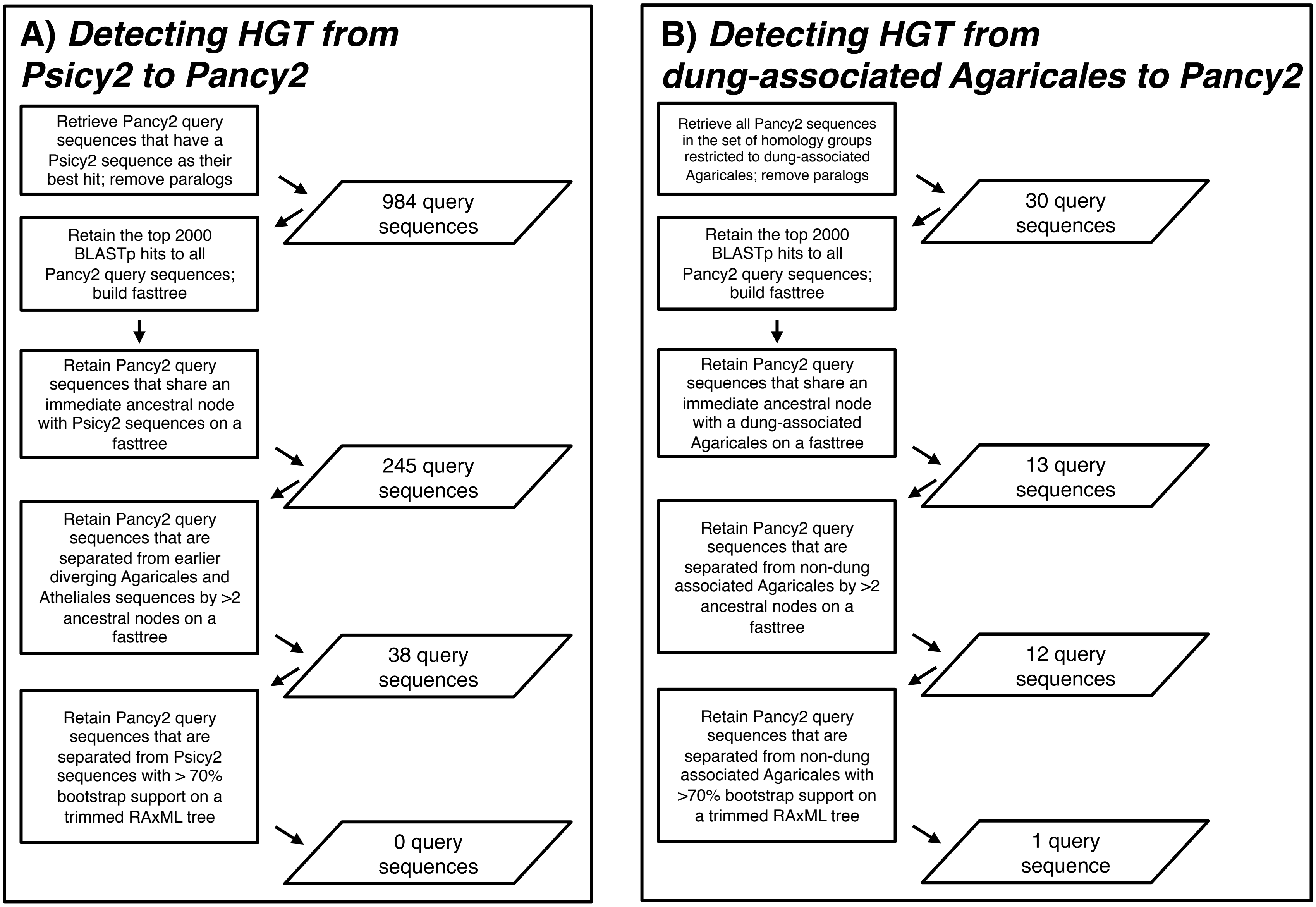
A) Detecting HGT from Psicy2 to Pancy2
Retrieve Pancy2 query sequences that have a Psicy2 sequence as their best hit; remove paralogs
984 query sequences
Retain the top 2000 BLASTp hits to all Pancy2 query sequences; build fasttree
Retain Pancy2 query sequences that share an immediate ancestral node with Psicy2 sequences on a fasttree
245 query sequences
Retain Pancy2 query sequences that are separated from earlier diverging Agaricales and Atheliales sequences by >2 ancestral nodes on a fasttree
38 query sequences
Retain Pancy2 query sequences that are separated from Psicy2 sequences with > 70% bootstrap support on a trimmed RAxML tree
0 query sequences
B) Detecting HGT from dung-associated Agaricales to Pancy2
Retrieve all Pancy2 sequences in the set of homology groups restricted to dung-associated Agaricales; remove paralogs
30 query sequences
Retain the top 2000 BLASTp hits to all Pancy2 query sequences; build fasttree
Retain Pancy2 query sequences that share an immediate ancestral node with a dung-associated Agaricales on a fasttree
13 query sequences
Retain Pancy2 query sequences that are separated from non-dung associated Agaricales by >2 ancestral nodes on a fasttree
12 query sequences
Retain Pancy2 query sequences that are separated from non-dung associated Agaricales with >70% bootstrap support on a trimmed RAxML tree
1 query sequence
